# *Moringa oleifera* Leaf Supplementation as a Glycemic Control Strategy in Subjects with Prediabetes

**DOI:** 10.3390/nu14010057

**Published:** 2021-12-24

**Authors:** Sonia Gómez-Martínez, Ligia E. Díaz-Prieto, Iván Vicente Castro, César Jurado, Nerea Iturmendi, Maria Carmen Martín-Ridaura, Nuria Calle, María Dueñas, María J. Picón, Ascensión Marcos, Esther Nova

**Affiliations:** 1 Immunonutrition Research Group, Department of Metabolism and Nutrition, Institute of Food Science and Technology and Nutrition (ICTAN)—CSIC, C/Jose Antonio Nováis 10, 28040 Madrid, Spain; sgomez@ictan.csic.es (S.G.-M.); ldiaz@ictan.csic.es (L.E.D.-P.); i.vicente@ictan.csic.es (I.V.C.); maria.duenasm@gmail.com (M.D.); amarcos@ictan.csic.es (A.M.); 2 Cea Bermúdez Primary Health Care Centre, Madrid Health Service, C/Cea Bermúdez 10, 28003 Madrid, Spain; cesar.jurado@salud.madrid.org (C.J.); nerea.iturmendi@salud.madrid.org (N.I.); 3 Madrid-Health, Madrid City Hall, 28007 Madrid, Spain; martinrmc@madrid.es (M.C.M.-R.); callefn@madrid.es (N.C.); 4 Hospital Virgen de la Victoria de Málaga, Campus de Teatinos, S/N, 29010 Malaga, Spain; mjpiconcesar@gmail.com

**Keywords:** *Moringa oleifera*, prediabetes, glycemic control, food supplement, gut microbiota, gastrointestinal hormones, polyphenol-rich plant food

## Abstract

*Moringa oleifera* (MO) is a multipurpose plant with a high polyphenol content, which is being increasingly consumed to lessen the risk of chronic metabolic diseases such as Type 2 diabetes; however, scientific evidence from clinical trials is scarce. A double-blind, randomized, placebo-controlled, parallel group intervention study with MO leaves as a food supplement was conducted in subjects with prediabetes. They consumed six daily capsules of MO dry leaf powder (2400 mg/day) (MO, *n* = 31) or placebo (PLC, *n* = 34) over 12 weeks. Glycemia, appetite-controlling hormones and gut microbiota composition were studied. ANCOVA with the fixed factor “treatment” and the basal value as covariate was used to compare the change score between the groups. The results showed significant differences between groups in the rate of change of fasting blood glucose (FBG) and glycated hemoglobin (HbA1c), which showed opposite directions during the intervention, decreasing in MO and increasing in PLC. No different change scores were found between the groups in microbiota, hepatic and renal function markers or the appetite-controlling hormones measured. In conclusion, MO supplementation resulted in favorable changes in glycaemia markers compared to placebo in the subjects with prediabetes studied, suggesting that MO might act as a natural antihyperglycemic agent.

## 1. Introduction

The *Moringa oleifera* (MO) tree, commonly referred to as ‘Drumstick tree’, belongs to the Moringaceae family and is the best known and most widely used of the 13 species of the *Moringa* genus. It is a fast-growing perennial tree cultivated in tropical and subtropical areas and currently spreading to other regions, such as the Iberian Peninsula [1]. There are several reasons for this worldwide cultivation; it is a protein-rich leafy vegetable and an interesting source of fiber, potassium, calcium, magnesium, β-carotene and α-tocopherol, that also contains high polyphenol levels [1,2]. MO leaves, seeds and pods are used as food or food supplement with medicinal properties. Specifically, MO dry leaves and leaf extracts have been shown to exert numerous in vitro activities and in vivo effects, including the hypoglycemic effect [3]. Its therapeutic value as a cardioprotective, hepatoprotective, neuroprotective, anti-asthmatic, anti-tumor, antimicrobial, hypolipidemic, modulator of intestinal microbiota and antidiabetic agent derives from its phytochemical constituents such as alkaloids and polyphenols [4,5,6]. In addition, bioactivity has been proved for peptide fractions (i.e., inhibition of alfa-amylase and angiotensin converting enzyme-1 activities) [7], isolated polysaccharide fractions [8,9] or the isothiocyanate derivatives of characteristic glucosinolates [10,11].

Diabetes mellitus (DM) type 2 is a chronic disease of increasing prevalence, which, together with obesity, emerges as a consequence of rapid societal changes affecting dietary patterns and physical activity levels [12]. Improving diet and lifestyle is the best strategy against the progression of prediabetes into diabetes. However, 5 to 10% of patients with prediabetes turn to type 2 DM per year and there is not a pharmacological solution without side effects in the long term. On the other hand, regarding lifestyle interventions, compliance is always an issue.

Regarding the hypoglycemic or anti-diabetic effect of MO, a fairly high number of studies have been performed in animal models of hyperglycemia. They majorly show significant improvements in blood glucose, both fasting and in response to a glucose tolerance test [13]. The mechanisms of action include the normalization of the gene expression of enzymes involved in glucose metabolism resulting in the restoration of liver glycolytic activity and glycogen storage as well as reducing gluconeogenesis and improving insulin signaling [13]. In addition, or secondary to these, delayed gastric emptying, inhibition of intestinal glucose uptake, improved glucose uptake in muscle and liver and the action of some insulin-like proteins present in the MO plant could also contribute to the hypoglycemic effect [14]. Regarding clinical trials in humans, to our knowledge there are only eight published studies and with very variable designs. Four of them are studies with single dose administration and postprandial glucose curve measurements [15,16,17,18] and four had a longitudinal design of daily MO leaf administration [19,20,21,22]. The postprandial studies mainly showed significant or marginally [15] significant results both in patients with type 2 DM and healthy subjects [15,17,18]. Among the longitudinal administration studies, mainly performed in type 2 DM patients, three found a significant decrease either in fasting blood glucose (FBG) [19,21] or glycated hemoglobin (HbA1c) [20]. However, several limitations are found in these studies. Two of them were not blind [19,21], another one did not state the exact dose of MO administered with the tablets and different basal values of HbA1c were reported in the control and experimental groups [20]. Finally, the last study, not showing significant effects of MO on glycemia markers might have been a too short intervention with only 28 days duration [22]. Thus, it is difficult to reach consensus about the indication of MO as an adjuvant therapy in the prevention and treatment of DM and well-designed intervention studies in patients with prediabetes and DM are certainly needed. Thus, the aim of this work was to evaluate whether daily consumption of MO leaf improves glycemic control in subjects with prediabetes in a randomized and double-blind clinical trial of mid-term duration. Secondarily, the effect on gut microbiota composition, hepatic and renal function and hormones involved in appetite control were also assessed, as well as the possible interactions between the basal levels of these biomarkers and the observed effects of MO supplementation on glucose control.

## 2. Materials and Methods

### 2.1. Study Design

This study was designed as a double-blind, randomized, placebo controlled, parallel group clinical trial performed in patients with prediabetes supplemented with six capsules of dry MO leaf powder or placebo per day during 12 wk. Eligible participants were randomized using a simple block randomization of 1:1.

### 2.2. Study Participants

Subjects were included within the age of 40 to 70 y. who complied with the American Diabetic Association (ADA) criteria [23] for the diagnosis of prediabetes (HbA1c: 5.7–6.4%, or fasting glucose: 100–125 mg/dL, or 2 h glucose tolerance test: 140–199 mg/dL) and who had never used drugs for glycemic control. Exclusion criteria were: type 1 and type 2 DM (following ADA criteria [23]), kidney disease (glomerular filtration rate < 60), uncontrolled hypertension (SBP > 140 or DBP > 90), severe cardiovascular disease, autoimmune disease, VIH, serious gastrointestinal disease, cancer, mental disease, high liver enzymes (×2 normal range for GOT, GPT or GGT), alcohol abuse (≥30 doses/week), morbid obesity (Body Mass Index, BMI > 35 kg/m^2^), pregnancy, food supplements in the previous two months or drugs interfering with glucose tolerance (β-blockers, antidepressants, antibiotics, estrogens, etc.).

Subjects with prediabetes were recruited for this study in collaboration with primary care centers in the Madrid Region. Practitioners performed the initial screening and referred potential candidates to the research team for final recruitment. Patients were mainly screened at two primary care settings although advertisement was also used for dissemination of the study. Eighty-five subjects were screened by the research team and a total of 73 patients were recruited and randomized. Enrolled subjects started the study either within the Placebo (PLC, *n* = 35) or Moringa (MO, *n* = 38) treatment groups.

This study was conducted according to the guidelines laid down in the Declaration of Helsinki and the Spanish law 14/2007 on Biomedical Research. All procedures involving human subjects were approved by the Ethics Committee of the Puerta de Hierro-Majadahonda University Hospital and the Bioethics committee of the Spanish National Research Council (CSIC). Written informed consent was obtained from all subjects before entering the study.

### 2.3. Intervention

Capsules were supplied by a dietary supplement manufacture company based in the Mediterranean region of Spain, which is also proprietary of the MO organic cultivation. Capsules contained 400 mg of organic MO dry leaf powder. Nutrient composition and polyphenol content are presented in Table S1 according to analyses performed in an accredited (ISO 9001:2015) analytical laboratory. The microbiological analysis reported no presence of pathogens, in accordance with regulatory specifications. Opaque white capsules were used both for MO and PLC supplementation, the last one filled with 400 mg of microcrystalline cellulose.

Patients entering the study were instructed to take two capsules before starting each main food (breakfast, lunch and dinner), on all days for 12 weeks and not to make any other dietary changes or lifestyle changes for the length of the intervention. They were appointed for three visits in order to measure primary and secondary outcomes at baseline (0 wk.), at intermediate (6 wk.) and end-of-study visits (12 wk.). Compliance with capsule intake was recorded by the participants in a chart and they were also asked to bring the bottles with any remaining pills as a second checking. Compliance was good (93%) with no differences between PLC and MO groups. Only two people had roughly 80% compliance in the MO group but all the rest had compliance above 87%.

All visits were programmed in the morning and the subjects provided a fecal sample collected one or two days ahead and kept frozen until delivered with cool transport to the research team. Following 12-h fasting, blood was withdrawn from the antecubital vein, which was collected in different vacutainer tubes for specific biomarker analysis.

### 2.4. Outcomes

The primary outcomes were the glycemic markers FBG and HbA1c. Secondary outcomes were serum insulin and gut hormones involved in appetite control, such as glucagon-like peptide 1 (GLP-1), ghrelin and peptide YY (PYY). Finally, nine representative groups or species of the human gut microbiota were analyzed in feces.

### 2.5. FBG and HbA1c Assessments

Blood was centrifuged at 1300× *g* during 15 min. within two hours after collection and several plasma aliquots were kept at −80 °C until analysis. Glucose was measured in serum by the hexoquinase method and HbA1c in EDTA plasma by ion exchange chromatography. Both were analyzed on the day in freshly collected samples in an accredited medical diagnostics laboratory (Unilabs Madrid S.A., certified by norm 9001:2008).

### 2.6. Assessment of Secondary Outcome Measures

Insulin was measured by chemo-luminescence in the same accredited diagnostics laboratory. Gut hormones were analyzed at ICTAN laboratory by xMAP Luminex^®^ technology with the Human Metabolic Hormone magnetic bead panel (Merck Millipore, Burlington, MA, USA) and read on Magpix 100 equipment (Luminex Corporation, Austin, TX, USA). To prevent the degradation of active ghrelin and GLP-1, one blood aliquot of 1 mL was treated before centrifugation with the protease inhibitors AEBSF (1 mg/mL final concentration) and DPP-IV inhibitor (10 µL per 1 mL blood) (Merck KGaA, Darmstadt, Germany). The sensitivity (minimum detectable concentration) of every analysis was as follows: Ghrelin: 13 pg/mL; PYY: 28 pg/mL; GLP-1: 1.2 pg/mL.

The gut microbiota was analyzed in fecal samples collected in sterile containers one or two days before each visit. Samples were immediately frozen at −20 °C and transported on the next day in refrigerated conditions to the study center where they were stored at −80 °C until analyses. Starting from 180–220 mg of each fecal sample, bacterial DNA was extracted using an optimized protocol as described in Gonzalez-Zancada et al. [24]. DNA was finally recovered with the commercial QIAamp DNA Fast Stool Mini Kit (QIAGEN GmbH, Hilden, Germany), following the manufacturer’s instructions and quantified using Nanodrop ND-1000 spectrophotometer (NanoDrop Technologies, Wilmington, DE, USA).

SYBR-Green real time PCR (qPCR) was performed for the detection of 16 S rRNA genes with specific primers (Table S2) targeted to the following bacterial groups: *Bacteroides*, *Blautia coccoides-Eubacterium rectale* group, *Clostridium* cluster IV group, *Bifidobacterium* spp., *Lactobacillus* spp., *Enterobacteriaceae*, *Enterococcus* spp. and the species *Faecalibacterium prausnitzii* and *Akkermansia muciniphila*. A standard curve for each qPCR assay was used for the quantification of target bacterial DNA in optimized amounts of fecal DNA samples. The detailed protocol was firstly described by Redondo et al. [25]. qPCR experiments were carried out in a Stratagene Mx3000P equipment (Agilent Technologies). The quality of the qPCR runs was assessed by the reaction efficiency, measured by the slope of the standard curve (r = −3.3 to −3.6) and non-template controls.

### 2.7. Diet and Anthropometry Assessments

Prior to each of these visits, patients received a three-day dietary registry form and were orally instructed on how to fill it in with as much detail as possible. This dietary registry was then reviewed and discussed with the participant upon collection to avoid missing relevant information on meals, ingredients or quantities. Anthropometrical measurements taken to the participants included weight, height, waist and hip circumferences and bioimpedance analysis (InnerScan BC-545; TANITA). All measurements were taken barefoot and with standard methods. Height was measured only at basal visit with a stadiometer (0.5 cm precision) and weight at each visit with the TANITA scale at the nearest 0.1 kg precision. BMI was calculated as Weight (kg)/Height (m)^2^. Circumferences were measured with an inelastic tape (SECA, precision 0.5 cm) by standard procedures.

### 2.8. Sample Size

Sample size calculation was based in published data [21] from a supplementation study with MO leaf during 10 wk. performed in postmenopausal women. Fasting blood glucose (FBG) was reported as Mean ± SE in that article and SD was calculated to be used in GPower 3.1. A within-group difference calculation was performed with one tail for the treated group, assuming that no change will occur in the placebo group, which, considering a 0.80 power and a 0.05 significance level resulted in a sample size of 66, which was increased by 10% to cover for potential drop-outs.

### 2.9. Randomization

The randomization was done using a computer-generated random number sequence. To ensure allocation concealment containers were labelled A or B and only one person (non-researcher), supplied the corresponding containers to the participants once the intervention had been assigned using the randomization sequence. All researchers involved in the study remained unaware of the bottles content. At 60% and 90% recruitment completion, equilibrium for sex and age between allocation groups was checked.

### 2.10. Postprandial Study

Previous to the 12-wk intervention study a postprandial study was carried out to test different doses of MO and choose one on the basis of its effects on subjects’ glycemia. Three patients with prediabetes were recruited for this study following the ADA criteria mentioned above. A standard breakfast (bread, ham, tomato sauce, milk and fresh apple, providing 500 kcal) was given to participants, plus, respectively, 1 PLC capsule, or 1, 3 or 6 capsules containing 400 mg of MO dry leaf powder on 4 consecutive visits, one week apart. The basal and postprandial (30, 60, 90, 120, 150 and 180 min) glucose and insulin values were measured and area under the curve (AUC) for glucose was calculated.

### 2.11. Statistical Analysis

Data sets were analyzed using IBM SPSS Statistics version 26. The data are expressed as the mean ± standard deviation (SD). Prior to data analyses, the distribution of the variables was checked through Kolmogorov-Smirnov test and box-plot representation and only gut hormones (ghrelin and GLP-1), total bilirubin and transaminase enzymes did not fit the normal distribution. For these variables, the normal distribution was obtained after log transformation.

General linear models (GLM) with repeated measures were used to analyze the effect of treatment and visit (within-subject factor) as well as their interaction (“treatment × visit”) to check whether the two groups had a different evolution along the time. In addition, to assess the effect of the MO supplementation, comparison of the change score between the groups in the main glycemic markers, FBG and HbA1c was performed.

The rate of change in serum/plasma biomarkers and in the microbiota was calculated with the formula:

[(value 12 wk − value 0 wk)/value 0 wk] × 100(1)

The difference between the groups in the variable’s rate of change was assessed by ANCOVA with the fixed factor “treatment” and the basal value as covariate.

In order to assess the influence of potential determinants of treatment response in the study subjects, an exploratory analysis of the difference in the basal levels of anthropometrical parameters, blood biomarkers and gut microbiota bacterial groups’ abundance between subject improving and not improving HbA1c in both treatment groups was carried out. For BMI and waist circumference, ANCOVA adjusted by sex was performed. Regarding the rest of the biomarkers, univariate ANCOVA with BMI adjustment was used with variables fitting a normal distribution and the Mann Whitney U test for those not fitting the normal distribution.

In the postprandial study, the glucose area under the curve (AUC) was calculated and compared for the different MO doses vs. placebo using the Wilcoxon test.

## 3. Results

**Figure 1:**
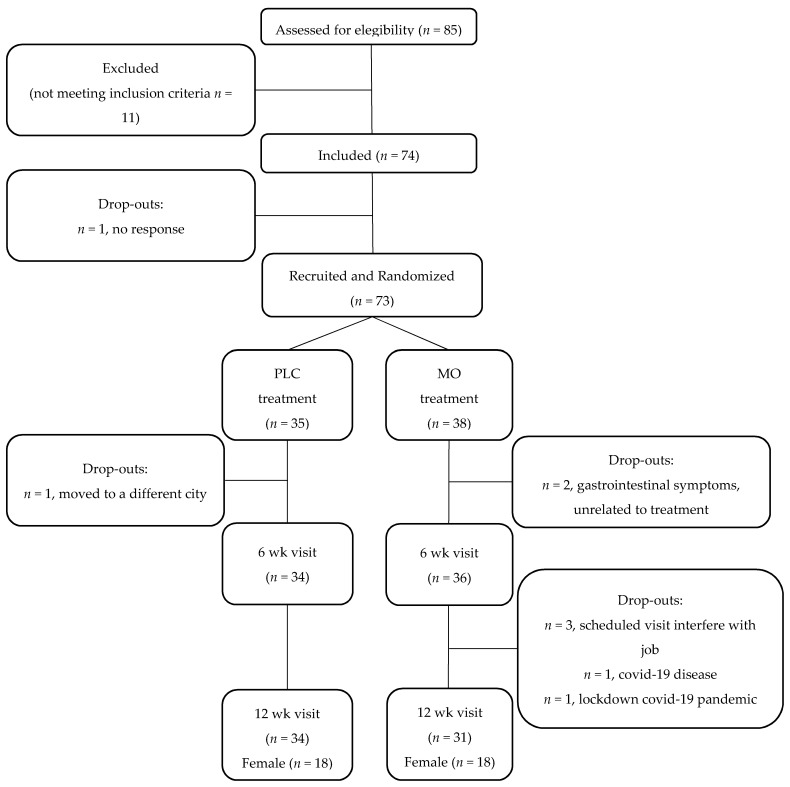
Flow chart of study subjects. PLC, Placebo; MO, *Moringa oleifera*; wk, weeks.

A flow chart of participants is presented in Figure 1. Sixty-five subjects finished the study, 34 in PLC (18 female) and 31 in MO (18 female). The demographic and anthropometrical characteristics of the participants are presented in Table 1. No differences were observed in the sex proportions, age or anthropometrical values between the subjects in the MO and PLC groups. The intervention did not modify the anthropometrical measures of the subjects in any of the groups (Table S3).

### 3.1. Dose Study

In the postprandial study, trends (*p* ≈ 0.1) were found for lower glucose AUC values with the 1 and 6 MO capsules doses tested compared with PLC (Figure S1). These findings were considered sufficiently relevant taking into account the limited number of subjects. Thus, six capsules were the election dose for the 12-wk. study.

### 3.2. Glycemic Control

No differences were observed in the basal values of glycemic variables between the subjects in both treatment groups. The intervention led to significant effects as shown by the significant treatment × visit interactions observed in all the glycemia related biomarkers and the significant differences between groups in the rate of change of FBG and HbA1c (Table 2), which showed opposite directions during the intervention, decreasing in MO and increasing in PLC. Insulin increased in both groups at the end of the study and no differences were observed in the rates of change between both groups. Similarly, the rate of change of the HOMA-IR was not different between the groups.

The treatment dependent changes in glycemia did not lead to a different proportion of subjects moving from the prediabetic condition to a normal glycemia after the intervention. A similar normal prediabetes ratio was observed in the MO and PLC groups after the intervention based in the fasting glucose and HbA1c values. Both groups showed around 20% of subjects converting to normal status (Table 3).

### 3.3. Secondary Outcomes Analyses

No significant differences were found in the rates of change of gut microbiota groups (Table 4), gut hormones (Table 5) and renal and hepatic function markers (Table 6). In addition, no significant treatment × visit interactions were found except for the *Enterococcus* spp. in microbiota composition and GOT hepatic enzyme, which further analyzed with within group comparisons did not reveal significant differences between 0 wk and 12 wk in any group (PLC or MO).

In the gut hormones analyzed, a generalized decreasing pattern was found independently of the treatment for all gut hormones (all “visit” effects were *p* < 0.05) (Table 5).

### 3.4. Prediction of HbA1c Improvement by Basal Biomarker Levels

The difference in basal anthropometrical parameters between subject improving and not improving HbA1c during the intervention was analyzed in the PLC and MO groups, respectively. No significant differences were found in basal BMI or waist circumference values between subjects improving or not improving HbA1c in any of the groups (PLC or MO).

**Figure 2:**
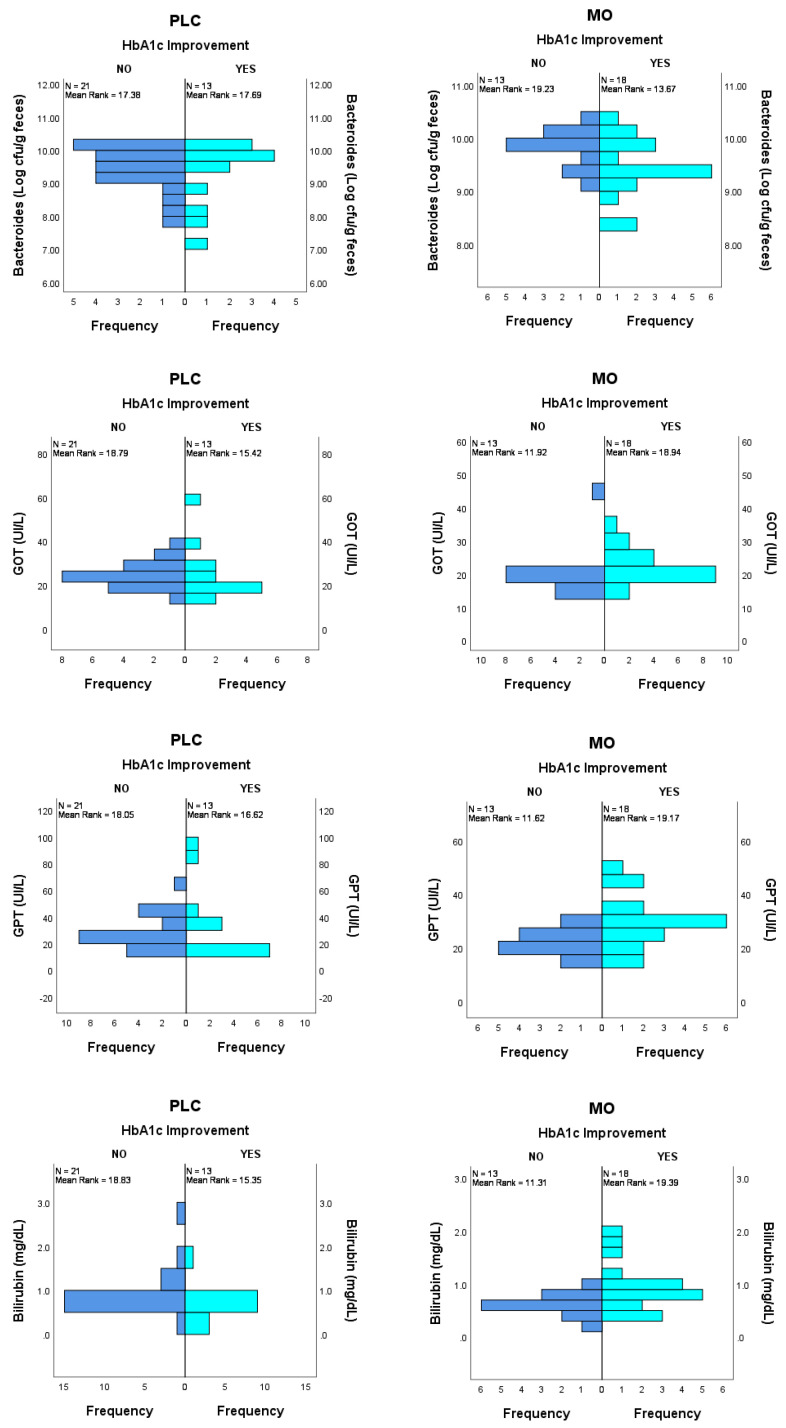
Potential predictor variables between subjects improving and not improving HbA1c during the intervention in the MO group. *Bacteroides*, ANCOVA with BMI adjustment, *p* = 0.085; GOT, GPT and Bilirubin, Mann Whitney U test, *p* = 0.032 *p* = 0.022 and *p* = 0.014, respectively. No differences were found in basal values in the PLC group in those variables.

Regarding basal bacterial group’s abundance, in the PLC group, no differences were found between subjects improving (*n* = 13) and not improving (*n* = 21) HbA1c along the 12-wk study. In the MO group, basal *Bacteroides* abundance showed a trends to be different in subjects improving HbA1c during the intervention (*n* = 18) compared to subjects whose HbA1c levels did not improve (*n* = 13) (9.45 ± 0.53 vs. 9.79 ± 0.37 CFU (Log)/g feces; *p* = 0.058 and *p* = 0.085 when adjusted by BMI) (Figure 2). No other differences were observed in bacterial groups.

For gut hormones, almost significant differences in basal levels of PYY and GLP-1 were found in the PLC group comparing subjects that improved and not improved HbA1c. For PYY, median and IQR values were 10.0 (10.0–51.25) vs. 43.6 (19.1–60.5) with a *p*-value of 0.055; for GLP-1, values were 3.44 (1.46–6.72) vs. 5.63 (3.22–9.02) with a *p*-value of 0.070. On the other hand, no differences were found in the gut hormone levels of subjects belonging to the MO group.

Regarding parameters of renal and hepatic function, significantly higher values of total bilirubin and the transaminase enzymes GOT and GPT were found at basal time in the MO group subjects with improved HbA1c compared to those without improved HbA1c. For total bilirubin, median and IQR values were 0.80 (0.58–1.05) vs. 0.50 (0.45–0.70) with a *p*-value of 0.014; for GOT, values were 21.5 (19.0–25.5) vs. 19.0 (16.5–21.0) with a *p*-value of 0.032 and for GPT, values were 28.5 (22.5–33.0) vs. 21.0 (20.0–25.0) with a *p*-value of 0.022. No differences were found in any of these renal and hepatic function biomarkers in the PLC group.

## 4. Discussion

This randomized placebo-controlled clinical trial designed to test the hypoglycemic effect of MO leaves in subjects with prediabetes showed different effects on glycemic variables in both treatment groups (PLC and MO), providing evidence of the potential use of MO as a glucose control agent in individuals with above normal blood glucose. Different changes were observed between groups in FBG and HbA1c after the 12-wk intervention, decreasing in MO and increasing in PLC group. As a result, 58% of subjects improved HbA1c levels after 12 wk. compared to baseline in the MO group and only 38% in the PLC group. A human study previously performed in type 2 diabetic patients reported a 5% decrease in HbA1C with 2 tablets of MO leaves consumed per day (from 7.81 ± 0.51% to 7.40 ± 0.63%; *p* < 0.01) [20]; however, the exact amount of MO per tablet was not specified and basal differences were observed between MO and control groups in initial HbA1c, which decreases somehow the resulting evidence grade since the subjects under MO treatment started with a worse glucose control than the other group.

In another study, 60 postmenopausal, but otherwise healthy women, divided into two parallel groups, received either no supplement or 7 g of MO leaves powder respectively, over 3 months [21]. In this period, 13.5% decrease in fasting glucose was observed. Moreover, the number of women normalizing glucose values (i.e., <110 mg/dL) in the MO group was higher than in the control group. This is a more pronounced effect on FBG than the one shown in our study. We found a 2.7% decrease in the MO group between basal and end-of-study time points and the same number of patients normalizing FBG (i.e., <100 mg/dL) in the MO and PLC groups. This might be due to the difference in the amount of MO dry leaves consumed in both studies which was three times higher in Kushwaha et al. [21]. Despite this, the significant effect observed on FBG change between the groups in the current study adds to the positive effects on HbA1c described above. Other studies have also reported significant effects of MO supplementation on postprandial glucose [19,20]. Another randomized-placebo controlled study failed to find improvements in any glycemia control marker in therapy-naïve DM patients undergoing a 28-day therapy with eight daily capsules of either MO (4 g/d) or placebo [22].A non-significant reduction of 3–4% of initial values was found in HbA1c as compared to baseline in both treatment arms, which reflected that self-monitoring plasma glucose provides feedback that improves glycemic control through lifestyle changes.

In the current study, insulin increased in both, the MO and PLC groups and no differences between groups were observed in their respective change score at the end of the study. Increased insulin secretion might effectively decrease glycemia levels or not, depending on the grade of insulin resistance. The literature is inconsistent regarding insulin observations following MO consumption. Four grams of MO leaves, in a single administration in healthy subjects, showed an increase in insulin secretion compared to placebo [16]. The AUC of the insulin/glucose ratio was 74% higher. An enhancing effect of insulin secretion has been reported in other MO studies [26,27]. This increased insulin secretion may be an advantage in patients with prediabetes that have still not developed insulin resistance. In addition, since a similarity has been reported between proteins isolated from MO leaf and insulin [28], the increase in insulin secretion might be explained by cross-reactivity of the antibodies used in the immunoassay with these MO proteins or the peptides resulting from their gastrointestinal digestion. Thus, all these considered, it is difficult to make a clear interpretation of the observed insulin results.

Regarding other potential mechanisms to explain the attenuation of hyperglycemia with MO, it has been suggested that herbal extracts [29] and specifically an ethanolic extract of MO leaves can inhibit the activity of intestinal α-glucosidase, which was mainly attributed to flavonoids in MO [30]. Some screened phytochemicals undergoing molecular docking and pharmacophoric studies have also revealed stable binding to the active binding pocket of the mutated insulin receptor, showing that they can be used as potential therapeutic drug candidates against DM [31].

The fact that we observed only marginally significant effects on HbA1c and glycemia comparing MO and PLC groups might be attributed to the small amounts of some of the bioactive compounds that would be available from the 2.4 g of MO administered with the capsules. This amount is lower than the dose used in animal experiments after conversion [13,32,33]. The lack of effect in the study of Taweerutchana et al. [22] in patients with diabetes who had a nine-point self-monitorization of fasting and postprandial glucose levels evaluated, have also been partially attributed to the low levels of compounds such as moringin or chlorogenic acid contained in the 4 g of MO dried leaf powder consumed daily. In addition, in that study, the 28-day duration of the intervention might have been too short. On the other hand, the fact that the mean HbA1c level in our subjects (5.8%) is near normal values could also hamper the finding of a more meaningful effect.

MO consumption in our study did not lead to relevant changes in the gut microbiota. Only one human study of MO supplementation studying microbiota effects was found in the published literature [34]. In this study, the influence of probiotic yogurt containing *Lactobacillus rhamnosus* GR-1 and 4 g MO leaf on the microbiota at different body sites was evaluated in pregnant women in Tanzania. No changes were observed in the women microbiotas. The stability of the gut microbiota seems to be maintained when MO dried leaves are consumed in small amounts of 2–4 g per day. On the other hand, this study did not find solid evidence that the inter-individual variability in the gut microbiota composition might influence the effect of MO on glycemia control.

MO supplementation did not have any effect on the gut hormones analyzed and no prior articles described effects of MO on appetite-controlling hormones. On the other hand, the decreasing pattern of the hormone levels observed in both MO and PLC groups seems unrelated to dietary changes since no differences have been noted in nutrient intakes during the intervention in any of the groups (Table S4). Thus far, it is not possible to explain the decrease in gut hormones observed in this study, but a very significant correlation was found between the change in ghrelin and the change in HbA1c during the intervention (Pearson’s coefficient = 0.412; *p* = 0.001) implying that ghrelin decrease was related to glucose control improvement, which is consistent with reported hyperglycemic and insulin resistance promoting effects of acyl ghrelin [35].

The consumption of MO did not alter hepatic or renal function, thus, indicating no damaging side effects of the supplementation. At the level used in this study it is not expected that long term consumption might induce a cumulative toxic effect on kidney or liver associated with the anti-nutrient compounds that are originally present in the leaves, such as saponins, tannins, terpenes or alkaloids [36]. Toxicity studies performed in animals have shown no histopathological changes in these organs with levels of consumption as high as 30 times that of a typical dose for humans [37]. On the other hand, the fact that higher basal levels of bilirubin and hepatic transaminase enzymes facilitated the effect of MO on improvement of HbA1c levels might be related to the hepatoprotective effects demonstrated for MO, as shown in diabetes-induced animal models [3,38].

The authors are aware that this study has several limitations. First, the analyses performed (ANCOVA), although showing a significant effect of the intervention on the main outcome, have only a small power due to the variability of the individual results and thus more studies would be necessary to ascertain the effectiveness with bigger sample size. However, in a within-group (0–12 wk) analysis the power raised to 0.74 for HbA1c in the MO group (results not shown), but remained low for FBG. In the same line, the very moderately raised mean glycaemia prior to the intervention in the sample studied might have prevented the finding of more significant effects. Another limitation refers to the exploratory nature of the analysis of the potential factors influencing the response to the intervention, which were not powered adequately in the conception of the investigation. The results shown here regarding the role that basal characteristics of hepatic function or even the microbiota might have on glycemic control by MO, do not allow solid conclusions and warrant confirmation. Despite these limitations, the current study provided evidence of MO effects on glycaemia control compared to a placebo intervention, while being superior to previous interventions which did not have a blind design [19,21] or had intervention and control groups with basal differences [20] or had a too short duration [22]. Future trials should investigate parts of MO other than the leaves, such as the seeds and roots, for activity.

In conclusion, this nutritional intervention study with MO supplementation resulted in favorable changes in glycaemia blood markers compared to placebo in the subjects with prediabetes studied, suggesting that MO plant might act as a natural anti-hyperglycemic agent, before any pharmacological treatment is prescribed.

## Figures and Tables

**Table 1:** Basal demographic and anthropometric characteristics.

	PLC (*n* = 34)	MO (*n* = 31)	*p*
Male:Female ^#^ (*n*, %)	16/18 (47/53)	13/18 (42/58)	0.678
Age (y)	56.1 ± 10.8	56.2 ± 9.2	0.967
Weight (kg)	79.2 ± 13.0	79.5 ± 14.1	0.928
Height (cm)	166.5 ± 10.0	164.4 ± 10.8	0.421
BMI	28.6 ± 3.8	29.4 ± 4.0	0.408
^1^WC (cm)			
Male	95.5 ± 11.2	101.3 ± 9.7	0.161
Female	92.3 ± 11.0	94.1 ± 8.7	0.581

Independent samples T test or ^#^ Chi square test. ^1^WC, waist circumference; PLC, Placebo; MO, *Moringa oleifera*.

**Table 2:** Glycemic control biomarkers in patients with prediabetes of the PLC and MO groups during the intervention.

		0 wk	6 wk	12 wk	*p* ^#^	Rate of Change 0–12 wk
Glucose (mg/dL)	PLC	104.2 ± 15.7	104.5 ± 18.0	106.2 ± 18.0	0.014	2.1 ± 9.9
	MO	103.4 ± 10.8	106.9 ± 8.4	100.6 ± 8.7		−2.3 ± 8.6
						*p*^Φ^ = 0.049; Power = 0.51
HbA1c (%)	PLC	5.83 ± 0.37	5.85 ± 0.42	5.87 ± 0.47	0.030	0.7 ± 4.2
	MO	5.88 ± 0.31	5.90 ± 0.35	5.79 ± 0.37		−1.5 ± 3.7
						*p*^Φ^ = 0.026 Power = 0.61
Insulin (µUI/mL)	PLC	8.74 ± 4.25	8.65 ± 2.80	10.56 ± 5.94	0.007	27.1 ± 57.7
	MO	11.11 ± 5.21	13.46 ± 6.01	12.37 ± 5.18		17.3 ± 31.5
						NS
HOMA-IR	PLC	2.26 ± 1.25	2.24 ± 0.82	2.83 ± 1.95	0.003	32.8 ± 78.3
	MO	2.86 ± 1.41	3.56 ± 1.58	3.10 ± 1.33		15.6 ± 34.0
						NS

Between-group differences were assessed with a GLM with within-subject factor “visit”, the fixed factor “treatment” and the interaction “treatment × visit”. The *p* ^#^-value corresponds to the “treatment × visit” factor. *p* ^Φ^, ANCOVA between treatment groups with basal value as covariate. NS, not significant.

**Table 3:** Number of patients moving from prediabetes to normal glycemia based on fasting glucose and HbA1c values at 12 wk.

	PLC, *n* (%)	MO, *n* (%)	*p* ^#^
Prediabetes	27 (79.4)	25 (80.6)	0.901
Normal	7 (20.6)	6 (19.4)	

*p*^#^, Comparison between treatment groups by Chi-square test.

**Table 4:** Microbiota (log cfu/g feces) in patients with prediabetes of the PLC and MO groups during the intervention.

		0 wk	6 wk	12 wk	*p* ^#^	Rate of Change 0–12 wk (*p* ^Φ^)
*Blautia coccoides- Eubacterium rectale*	PLC	11.56 ± 0.29	11.61 ± 0.45	11.75 ± 0.29	0.218	1.7 ± 3.3
	MO	11.63 ± 0.32	11.72 ± 0.39	11.70 ± 0.38		0.7 ± 3.6
						NS
*Bacteroides fragilis* group.	PLC	9.38 ± 0.80	9.39 ± 0.84	9.42 ± 0.67	0.482	0.6 ± 5.5
	MO	9.59 ± 0.49	9.61 ± 0.57	9.58 ± 0.58		−0.1 ± 4.5
						NS
*Clostridium cluster* IV	PLC	8.39 ± 0.36	8.38 ± 0.58	8.49 ± 0.36	0.613	1.2 ± 5.2
	MO	8.43 ± 0.33	8.47 ± 0.33	8.47 ± 0.39		0.5 ± 4.8
						NS
*Bifidobacterium* spp.	PLC	7.76 ± 0.40	7.85 ± 0.47	7.90 ± 0.39	0.697	1.9 ± 4.8
	MO	7.78 ± 0.39	7.85 ± 0.40	7.83 ± 0.41		0.9 ± 5.8
						NS
*Enterobacteriaceae*	PLC	6.59 ± 1.15	6.56 ± 0.96	6.64 ± 1.02	0.404	3.5 ± 22.5
	MO	6.18 ± 0.86	6.42 ± 1.07	6.57 ± 0.90		7.7 ± 17.5
						NS
*Enterococcus* spp.	PLC	4.74 ± 1.02	5.14 ± 1.09	5.04 ± 1.0	0.016	9.1 ± 23.1
	MO	4.89 ± 0.97	4.62 ± 0.80	4.87 ± 0.78		2.5 ± 21.9
						NS
*Lactobacillus* group	PLC	4.34 ± 1.15	4.49 ± 1.06	4.67 ± 0.99	0.091	11.8 ± 26.9
	MO	4.37 ± 0.80	4.05 ± 0.74	4.33 ± 0.70		1.0 ± 18.6
						NS
*Faecalibacterium prausnitzii*	PLC	9.33 ± 0.36	9.38 ± 0.39	9.46 ± 0.35	0.438	1.5 ± 4.4
	MO	9.42 ± 0.30	9.49 ± 0.35	9.46 ± 0.38		0.5 ± 4.0
						NS
*Akkermansia muciniphila*	PLC	7.34 ± 1.25	6.99 ± 1.58	7.18 ± 1.43	0.885	−1.3 ± 16.7
	MO	7.19 ± 1.26	6.69 ± 1.33	6.96 ± 1.56		−0.5 ± 27.0
						NS

Between-group differences were assessed with a GLM with within-subject factor “visit”, the fixed factor “treatment” and the interaction “treatment × visit”. The *p* ^#^-value corresponds to the “treatment × visit” factor. *p* ^Φ^, Comparison between treatment groups by independent sample T test. NS, not significant.

**Table 5:** Gut hormones in patients with prediabetes of the PLC and MO groups during the intervention.

		0 wk	6 wk	12 wk	GLM *p* ^#^	Rate of Change 0–12 wk (*p* ^Φ^)
Ghrelin (pg/mL)	PLC	141.7 ± 91.2	128.6 ± 80.4	99.1 ± 70.6	0.997	−30.7 ± 25.7
	MO	134.5 ± 92.4	121.8 ± 76.6	87.5 ± 55.8		−30.8 ± 26.2
						NS
PYY (pg/mL)	PLC	37.4 ± 26.5	38.5 ± 26.5	30.1 ± 23.7	0.161	−10.3 ± 36.0
	MO	34.0 ± 20.4	29.1 ± 22.2	28.0 ± 23.2		−1.8 ± 79.1
						NS
GLP-1 (pg/mL)	PLC	6.8 ± 6.6	6.6 ± 7.7	5.4 ± 5.2	0.876	18.4 ± 135.8
	MO	7.2 ± 5.6	6.2 ± 4.8	6.4 ± 6.8		6.1 ± 110.9
						NS

General linear model (GLM) with within-subject factor “visit”, the fixed factor “treatment” and the interaction “treatment × visit”. The *p* ^#^-value corresponds to the “treatment × visit” factor. *p* ^Φ^, Comparison between treatment groups by independent sample T test (Ghrelin) or Mann-Whitney U test (PYY and GLP-1). NS, not significant; PYY, peptide YY; GLP-1, glucagon-like peptide-1.

**Table 6:** Renal and hepatic function parameters in serum samples of patients with prediabetes of the PLC and MO groups during the intervention.

		0 wk	6 wk	12 wk	*p* ^#^	Rate of Change 0–12 wk (*p* ^Φ^)
Urea (mg/dL)	PLC	36.9 ± 7.4	35.6 ± 7.9	37.2 ± 8.7	0.400	2.9 ± 20.1
	MO	37.4 ± 6.7	38.5 ± 6.7	38.9 ± 7.4		4.8 ± 20.0
						NS
BUN (mg/dL)	PLC	17.2 ± 3.4	16.6 ± 3.7	17.4 ± 4.1	0.402	2.9 ± 20.1
	MO	17.5 ± 3.2	18.0 ± 3.1	18.1 ± 3.5		4.8 ± 19.9
						NS
Uric acid (mg/dL)	PLC	5.73 ± 1.07	5.40 ± 0.93	5.67 ± 0.92	0.291	0.0 ± 10.7
	MO	5.40 ± 1.45	5.40 ± 1.34	5.58 ± 1.34		3.3 ± 16.5
						NS
Creatinine (mg/dL)	PLC	0.78 ± 0.17	0.72 ± 0.16	0.77 ± 0.16	0.785	0.7 ± 15.7
	MO	0.76 ± 0.13	0.77 ± 0.13	0.76 ± 0.15		0.6 ± 12.2
						NS
Total Bilirubin (mg/dL)	PLC	0.78 ± 0.44	0.71 ± 0.37	0.75 ± 0.39	0.880	−0.1 ± 34.7
	MO	0.76 ± 0.41	0.69 ± 0.34	0.73 ± 0.42		−1.2 ± 32.8
						NS
GOT (UI/L)	PLC	25.7 ± 8.7	22.2 ± 6.0	23.4 ± 7.7	0.028	−3.1 ± 20.7
	MO	21.8 ± 6.4	23.7 ± 9.5	23.5 ± 7.0		11.1 ± 31.2
						NS
GPT (UI/L)	PLC	30.9 ± 19.1	27.0 ± 24.7	28.5 ± 14.8	0.339	−0.4 ± 26.4
	MO	26.4 ± 8.3	26.0 ± 9.8	26.8 ± 8.2		5.3 ± 32.4
						NS
GGT (UI/L)	PLC	36.9 ± 41.0	36.0 ± 50.8	35.6 ± 36.6	0.549	3.6 ± 19.4
	MO	30.4 ± 21.2	31.1 ± 20.4	29.1 ± 16.5		3.9 ± 30.7
						NS

General linear model (GLM) with within-subject factor “visit”, the fixed factor “treatment” and the interaction “treatment × visit”. The *p* ^#^-value corresponds to the “treatment × visit” factor. *p* ^Φ^, ANCOVA between treatment groups with basal value as covariate. NS, not significant; BUN, blood urea nitrogen; GOT, glutamic-oxaloacetic transaminase; GPT, glutamate-piruvate transaminase; GGT, gamma glutamyl transferase.

## Data Availability

The data presented in this study are available on request from the corresponding author, due to privacy restriction. The study has been registered in www.ClinicalTrials.gov (Identifier: NCT04734132).

## Supplementary Materials

The following are available online at https://www.mdpi.com/article/10.3390/nu14010057/s1, Figure S1: Postprandial glucose of three participants with prediabetes in the dose study, Table S1: Nutrient and polyphenol content in the MO dry leaf powder, Table S2: Primers used in qPCR gut microbiota analysis, Table S3: Anthropometrical values at basal and end-of-intervention time points, Table S4: Energy and nutrient intakes at the three study visits in each group.
