# Supplementary material for: Moringa oleifera Leaf Supplementation as a Glycemic Control Strategy in Subjects with Prediabetes
Source: Nutrients. 2021 Dec 24;14(1):57. doi: 10.3390/nu14010057 (PMC8746299; doi:10.3390/nu14010057)
Supplement: Supplementary file 1 [file nutrients-14-00057-s001.zip › nutrients-1459653-supplementary.pdf]

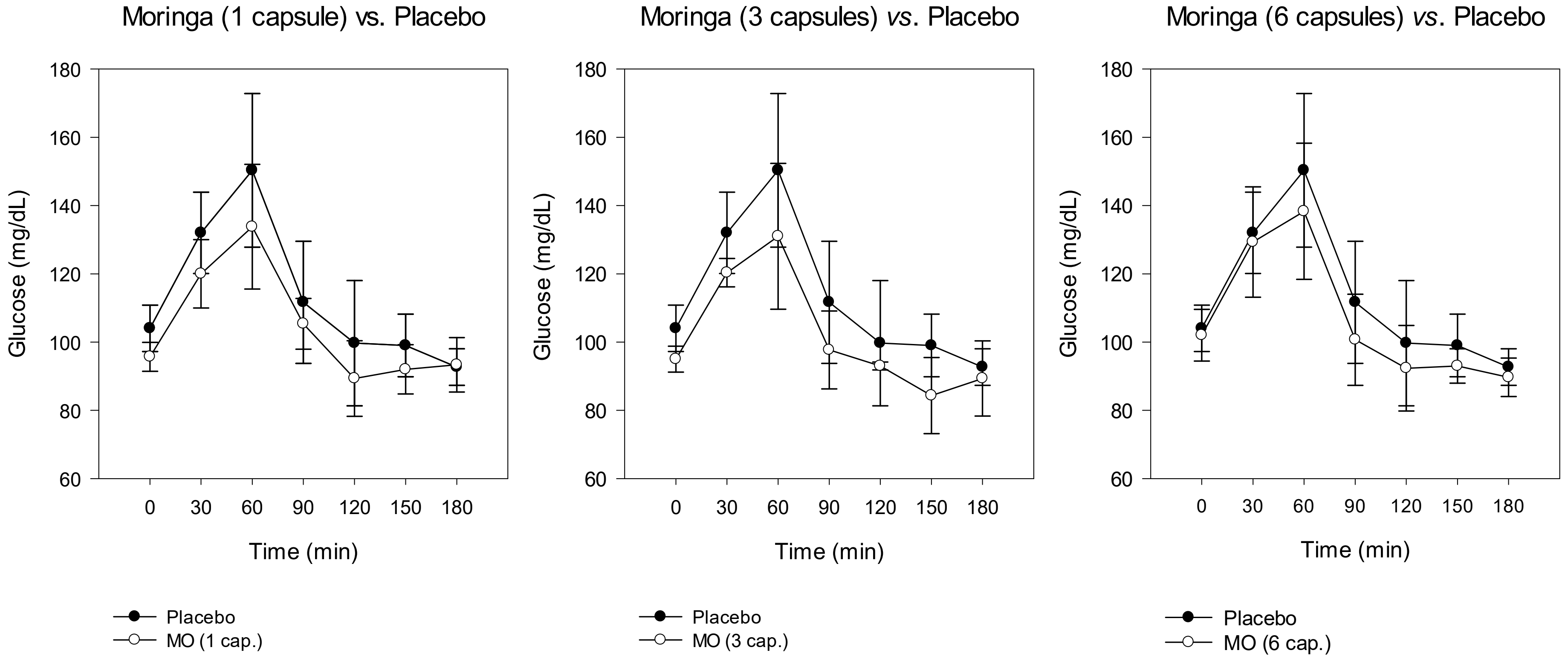

Figure S1. Postprandial glucose of three participants with prediabetes in the dose study. AUC was calculated with the trapezoidal rule and compared with that of the PLC group using Mann-Whitney U test. Non-significant trends (P≈0.1) were obtained with the doses of 1 and 6 capsules.

Table S1. Nutrient and polyphenol content in the MO dry leaf powder.

**NUTRITIONAL ANALYSIS**	
Energy (kcal/100 g)	350
Energy (kj/100 g)	1465
Fat (%)	4.66
Of which: saturated (%)	0.70
Carbohydrates (%)	49.27
Of which: sugars (%)	0.05
Proteins (%)	27.78
Salt (%)	0.71
**CHEMICAL ANALYSIS**	
Calcium (mg/100g)	4854
Potassium (mg/100g)	2761
Magnesium (mg/100g)	543
Sodium (mg/100g)	541
Phosphorus (mg/100g)	46
Iron (mg/100g)	21
Zinc (mg/100g)	8
Manganese (mg/100g)	5
Total polyphenols (ppm)	2300

Table S2. Primers used in qPCR gut microbiota analysis.

Bacteria	Primer	Sequence (5′→3′)	Size (pb)	Reference
Blautia coccoides-	gCcoc-F	AAATGACGGTACCTGACTAA	440	[1]
Eubacterium rectale	gCcoc-R	CTTTGAGTTTCATTCTTGCGAA		
Bacteroides fragilis	Bfra-F	ATAGCCTTTCGAAAGRAAGAT	495	[1]
group	Bfra-R	CCAGTATCAACTGCAATTTTA		
Clostridium cluster	sg-Clept-F	GCACAAGCAGTGGAGT	239	[1]
IV	sg-Clept-R3	CTTCCTCCGTTTTGTCAA		
Bifidobacterium spp.	BifF	TCGCGTC(C/T)GGTGTGAAAG	243	[2]
	BifR	CCACATCCAGC(A/G)TCCAC		
Enterobacteriaceae	Eco1457F	CATTGACGTTACCCGCAGAAGAAGC	195	[3]
	Eco1652R	CTCTACGAGACTCAAGCTTGC		
Enterococcus spp.	Enteroc_F	CCCTTATTGTTAGTTGCCATCATT	123	[2]
	Enteroc_R	ACTCGTTGTACTTCCCATTGT		
Lactobacillus group	Lacto_F	AGCAGTAGGGAATCTTCCA	200	[4,5]
	Lacto_R	CACCGCTACACATGGAG		
Faecalibacterium	Fprau223-F	GATGGCCTCGCGTCCGATTAG	199	[3]
praustnizzi	Fprau420-R	CCGAAGACCTTCTTCCTCC		
Akkermansia	F:S-St-Muc-1437-a-	CCTTGCGGTTGGCTTCAGAT	327	[6]
muciniphila	A-20	CAGCACGTGAAGGTGGGGAC		
	R: S-St-Muc-1129-			
	a-a-20			

Table S3. Anthropometrical values at basal and end-of-intervention time points.

	PLC				MO		
	0wk	12wk	$p^{\#}$		0wk	12wk	$p^{\#}$
Weight (*n* = 31)	78.2±12.2	78,5±12.1	0.465	Weight (*n* = 29)	80.1±14.1	79.6±13.3	0.319
BMI (*n* = 31)	28.4±3.8	28.5±3.7	0.436	BMI (*n* = 29)	29.7±3.9	29.5±3.9	0.328
WC				WC			
Male (*n* = 10)	92.1±8.2	91.0±7.7	0.263	Male (*n* = 6)	96.9±6.9	96.9±7.6	0.916
Female (*n* = 16)	92.0±11.7	92.7±11.0	0.479	Female (*n* = 13)	92.3±7.3	90.7±8.5	0.136

Mean±SD. [#]Paired T test for weight and BMI comparison and Wilcoxon Signed Rank test for WC. [1]WC, waist circumference. WC missing values are due to distancing security measures during the covid-19 pandemic in subjects finishing the study during lockdown.

Table S4. Energy and nutrient intakes at the three study visits in each group.

		0wk	6wk	12wk	$P^{\#}$
Calories (Kcal)	PLC	1888±562	1905±394	1966±433	NS
	MO	1893±497	1997±448	1978±542	
Protein (g)	PLC	80.2±21.0	86.4±22.8	87.6±20.2	P_{visit} = 0.007
	MO	80.6±22.9	88.5±17.3	88.4±23.6	
Protein (%)	PLC	17.32±3.00	18.22±3.68	18.03±3.03	NS (P_{visit} = 0.078
	MO	17.10±2.89	18.16±3.54	18.10±3.20	
Carbohydrates (g)	PLC	161.4±54.7	157.2±44.0	167.2±43.0	NS
	MO	161.8±45.0	172.7±52.4	157.1±46.2	
Carbohydrates (%)	PLC	34.63±6.52	33.27±7.51	34.38±6.40	NS
	MO	34.47±6.00	34.31±6.00	34.30±6.43	
Lipids (g)	PLC	91.0±33.5	90.4±23.3	91.9±26.1	NS
	MO	91.7±27.3	92.2±22.8	94.3±31.3	
Lipids (%)	PLC	42.67±7.55	42.61±6.51	41.86±5.82	NS
	MO	43.61±5.72	41.97±7.05	42.62±7.41	
AGS (g)	PLC	26.2±11.4	27.7±8.8	27.9±9.1	NS
	MO	27.7±10.7	27.0±7.5	27.7±11.4	
AGS (%)	PLC	12.20±2.89	13.20±3.69	12.62±2.40	NS
	MO	13.00±2.66	12.20±2.39	12.35±2.99	
AGM (g)	PLC	42.6±16.9	42.11±12.0	42.2±13.6	NS
	MO	42.2±12.0	42.9±12.3	43.9±13.9	
AGM (%)	PLC	19.95±4.42	19.78±3.45	19.28±4.17	NS
	MO	20.27±3.61	19.66±4.79	20.06±4.03	
AGP (g)	PLC	13.1±6.0	12.3±5.1	12.5±5.4	NS
	MO	13.5±6.3	13.5±5.5	14.1±6.5	

AGP (%)	PLC	6.18±2.32	5.75±1.90	5.73±2.20	NS
	MO	6.42±2.58	6.13±2.02	6.30±2.21	
Cholesterol (mg)	PLC	298±110	353±142	355±105	NS
	MO	331±122	326±101	331±129	
Fiber (g)	PLC	22.6±8.0	21.6±6.9	19.5±5.7	NS
	MO	20.0±4.9	22.1±8.1	19.8±6.1	

Data presented are Mean±SD. GLM with the whitin-subjects factor "visit", the between-subjects factor "treatment" and the interaction "treatment*visit". $p^{\#}$, significant effect as indicated in the subscript.

References

1. Matsuki, T.; Watanabe, K.; Fujimoto, J.; Takada, T.; Tanaka, R. Use of 16S rRNA gene-targeted group-specific primers for real-time PCR analysis. of predominant bacteria in human feces. *Appl. Environ. Microbiol.* **2004**, *70*, 7220–7228. https://doi.org/10.1128/AEM.70.12.7220-7228.2004.
2. Rinttilä, T.; Kassinen, A.; Malinen, E.; Krogius, L.; Palva, A. Development of an extensive set of 16S rDNA-targeted primers for quantification of pathogenic and indigenous bacteria in faecal samples by real-time PCR. *J. Appl. Microbiol.* **2004**, *97*, 1166–1177. https://doi.org/10.1111/j.1365-2672.2004.02409.x.
3. Bartosch, S.; Fite, A.; Macfarlane, G.; McMurdo, M.E. Characterization of Bacterial Communities in Feces from Healthy Elderly Volunteers and Hospitalized Elderly Patients by Using Real-Time PCR and Effects of Antibiotic Treatment on the Fecal Microbiota. *Appl. Environ. Microbiol.* **2004**, *70*, 3575–3581. https://doi.org/10.1128/AEM.70.6.3575-3581.2004.
4. Heilig, H.G.; Zoetendal, E.G.; Vaughan, E.E.; Marteau, P.; Akkermans, A.D.; de Vos, W.M. Molecular diversity of Lactobacillus spp. and other lactic acid bacteria in the human intestine as determined by specific amplification of 16S ribosomal DNA. *Appl. Environ. Microbiol.* **2002**, *68*, 114–123. https://doi.org/10.1128/AEM.68.1.114-123.2002.
5. Walter, J.; Hertel, C.; Tannock, G.W.; Lis, C.M.; Munro, K.; Hammes, W.P. Detection of *Lactobacillus*, *Pediococcus*, *Leuconostoc*, and *Weissella* species in human feces by using group-specific PCR primers and denaturing gradient gel electrophoresis. *Appl. Environ. Microbiol.* **2001**, *67*, 2578–2585. https://doi.org/10.1128/AEM.67.6.2578-2585.2001.
6. Derrien, M. Mucin Utilisation and Host Interactions of the Novel Intestinal Microbe *Akkermansia muciniphila*. Ph.D. Thesis, Wageningen University, Wageningen, The Netherlands; With Summary in Dutch and French 2007. ISBN 978-90-8504-644-8.
